# Graduate Teaching Facilities in London Hospitals

**Published:** 1906-07-21

**Authors:** 


### GRADUATE TEACHING FACILITIES IN LONDON HOSPITALS.

### SEAMEN'S HOSPITAL (DREADNOUGHT), GREENWICH: THE LONDON SCHOOL OF CLINICAL MEDICINE.

By an oversight, pointed out to us by the Secretary of the Seamen's Hospital, Greenwich, we alluded to the London School of Clinical Medicine as the School of Tropical Medicine. To neutralise the effect of this we give another clinique from the London School of Clinical Medicine at Greenwich in illustration of the practical character of the surgical teaching as expounded by Mr. Lawrie McGavin, F.R.C.S., one of the surgeons attached to this important clinical London centre of graduate instruction. The notes are a *resumé* not of a dreary lecture, but of information drawn by the instructor from the practitioners in attendance by way of question and answer—the whole forming a fairly useful picture of the subject under discussion, and conveyed in a form likely to be retained by the graduates.

### NOTES ON THE INDICATIONS IN A CASE OF CONCUSSION OF THE BRAIN.

Text-books say that the clot which is due to true subconjunctival hæmorrhage from fracture of the base of the skull is darker in colour than that which is usually termed a black-eye. As a rule this gives but little help. One practical point is that in fractured base the discoloration is usually on the outer side, and rarely on the inner. Conjunctival hæmorrhage from a black-eye is very often punctate, and occurs in several places; in fracture of the base it is always single, and gradually comes forward.

#### PUPILS AND PULSE.

The patient was in a comatose condition when admitted into the hospital, and remained so without exhibiting any other signs of cerebral laceration except a slight irritability. In pure concussion the symptoms one expects are unconsciousness with pupils equal but contracted, or with only a very sluggish reaction besides the ordinary signs of shock —shallow breathing, pallor, and clamminess of skin, weakness of pulse. In concussion, anything in the pulse-rate under 20 is supposed to be incompatible with life. In this case it is as shallow as 30. When the patient was admitted the pulse was 72 and 80. As a rule, in a typical case of concussion it is 28, 30, 35. Another interesting point about the pulse is that it frequently intermits a beat. This is different from a compression pulse, which is distinctly more forcible, fuller, and no longer intermits. It becomes more and more regular and bounding, and increases in rate, and if the medulla is involved it will go to 100, 120, 130, or, even when the patient is moribund, up to 150 or 156—dying away as the cardiac centres become involved, becoming more rapid and feebler. It is the pulse that gives the first indication of the change from concussion into compression.

#### EYE CHANGES IN COMPRESSION.

If the concussion is deep the patient cannot be roused, or he will only make an abortive attempt to push the hand away. In concussion one would expect the reflexes to be exaggerated, the brain function being, as it were, cut off; but it is not so—they are abolished. The eye changes form, another symptom of the onset of compression. The pupils begin to dilate, but in a noteworthy manner. The eye changes are easily remembered if this formula is borne in mind. Say the patient is struck on the head at 3 o'clock P.M., and he begins to get compression in an hour's time, the first sign of compression, quite apart from the eyes, is the partial recovery of consciousness and a complaint of being sleepy. This is soon followed by a sound sleep, stertorous respiration, and paralysis of the soft palate. But one of the earliest changes is in the eye. Inequality of the pupils will be noted. On the side that starts first to dilate is the side of compression. The pupil then begins to contract, and gets smaller than ever until it becomes almost pin-point. By that time the eye on the sound side may start the same phenomena. About the time that both pupils become pin-pointed stertorous breathing has supervened. Then gradually they dilate again. When both pupils are dilated fully the chances are the patient will be beyond surgical help.

#### STERTOROUS BREATHING.

This stertorous breathing is due partly to paralysis of the buccinator. A good rule to remember the nerve-supply of the muscles of the face is that all muscles of expression are supplied by the facial nerve, and all muscles of mastication are supplied by the fifth. Muscles which are both are supplied by both nerves. The buccinator is the least important of the two sets of muscles in compression. The soft palate is the chief. It becomes paralysed and the patient snores, from the soft palate flapping to and fro. Its nerve-supply is the descending branches of Meckel's ganglion. It is the fifth nerve chiefly that is paralysed in compression.

#### TEMPERATURE.

The temperature is another important indication. In concussion it is either normal or subnormal. In this case the temperature is gradually rising. This rise may be due to meningitis or lepto-meningitis, abscess or thrombosis or embolism in connection with abscess. There may also be hæmorrhage into the pons or into the fourth ventricle, where the heat regulating apparatus is stored up. It may go up to 106° or 107° in fatal cases. In this case there is no hæmorrhage into the pons or fourth ventricle.

#### THE PULSE.

The pulse has become barely perceptible. The patient has had no fits of any sort, and no spasticity indicating irritation of the cortex. The temperature rise may be due to a certain degree of meningitis or local irritation. No localising symptoms whatever are present. The patient is practically moribund. Probably he has laceration of the brain, but the symptoms taken together point strongly to lepto-meningitis (inflammation of the pia and arachnoid membranes). Lumbar puncture has been performed, and half a test-tubeful of fluid without any micro-organisms withdrawn. No good could have been done by operation.

### Treatment.

With regard to the ordinary treatment of concussion, it is well to surround the patient with warm bottles, and to give a hypodermic injection of strychnine. There may be sufficient damage to produce hæmorrhage into the brain, and if brandy is administered the heart rhythm may increase, and bring about the very thing it is desirable to avoid. Brandy is rather dangerous in concussion. In nearly all hospitals the ice-cap is at once applied. It seems unreasonable that a man should be surrounded with hot bottles, and then that an ice-cap should be applied to his head. The idea is that the ice combats the hæmorrhage. But hæmorrhage is going to occur only when reaction sets in. The condition of the brain in concussion and sleep is anæmia (first pointed out by Durham), and that condition results in over-dilatation when reaction sets in and sets up hæmorrhage into the tissues round about. So that when the ice-bag is applied it still more increases the cerebral anæmia, and when reaction sets in later, the effect of the ice-bag is lost through having been used too soon, and the result is that the thing to be avoided has been produced. In using the ice-bag it should be recalled that the hæmorrhage is not going on into the substance of the brain, but on to the meninges. An ice-bag on the outside of the skull will cool the brain and diminish the surface circulation. As a rule, it is applied too soon in cases of concussion. It should be left out of account in the treatment until the patient is about to react and shows signs of returning circulation.

### The Urine.

Suppression of urine may occur in concussion. Drugs will not produce diuresis, and sudorifics tend to lower the man's condition. It is not safe to raise the blood-pressure. Caffein and such drugs of that kind may be given, but suppression is probably a fatal condition. Retention of urine should be relieved by a catheter. Constipation if of common occurrence in nearly every case. In this case the patient had large doses of calomel (grains x.) and doses of croton oil without any effect. An enema cleared the rectum.

### The Sequelæ.

An important question is that of the supervention of complications. After recovery from a severe concussion, say, lasting six weeks, and about which the friends of the patient should have warning, one condition sometimes left is Jacksonian epilepsy, dependent on local irritation of the meninges, due to thickening of the inner table of the skull. Other causes of this epilepsy are fibrous adhesion of the dura mater to the skull, possibly a depressed spicule of the inner table of the skull, or adhesion of the dura mater and the pia mater to the surface of the brain. There may be sclerosis of the brain itself, produced presumably either by laceration or hæmorrhage into the substance of the brain. This causes trouble by producing fibrous adhesions. The hæmorrhage may have been punctate and produced hæmatoid in crystals. There are other causes in which nothing is demonstrable to account for the epilepsy.

Another sequela of concussion in later life is vertigo, to such a degree that the patient is unable to go up a height or a ladder. These cases are of importance in the wage earning classes. They must be warned of this danger. A serious condition which is usual after concussion is increased susceptibility to the influence of alcohol. An old Army officer, who previous to his injury could carry his liquor like most people, after recovery did not dare to take liquor, but became boisterous even after a glass of wine. That condition is not uncommon. There may be mental deficiency manifested in lapse or loss of memory—a form of minor epilepsy—not, however, exhibiting the train of symptoms that minor epilepsy has. The mind for the time being becomes absolutely blank. This lapse or loss of memory may occur for things that happened a day or two previously to the accident. Not many years ago the cause of a railway accident (probably at Slough) was traced to the fact that long prior to the occurrence the driver had a blow on the head, and very occasionally suffered from these lapses of memory.

